# Regulation of adult stem cell function by ketone bodies

**DOI:** 10.3389/fcell.2023.1246998

**Published:** 2023-08-31

**Authors:** Ole Emil Andersen, Jens Vase Poulsen, Jean Farup, Antoine de Morree

**Affiliations:** ^1^ Department of Public Health, Aarhus University, Aarhus, Denmark; ^2^ Steno Diabetes Center Aarhus, Aarhus University, Aarhus, Denmark; ^3^ Department of Biomedicine, Aarhus University, Aarhus, Denmark

**Keywords:** adult stem cells, ketone bodies, stem cell function, regenerative medicine, tissue homeostasis, cellular metabolism, beta-hydroxybutyrylation

## Abstract

Adult stem cells play key roles in tissue homeostasis and regeneration. Recent evidence suggests that dietary interventions can significantly impact adult stem cell function. Some of these effects depend on ketone bodies. Adult stem cells could therefore potentially be manipulated through dietary regimens or exogenous ketone body supplementation, a possibility with significant implications for regenerative medicine. In this review we discuss recent findings of the mechanisms by which ketone bodies could influence adult stem cells, including ketogenesis in adult stem cells, uptake and transport of circulating ketone bodies, receptor-mediated signaling, and changes to cellular metabolism. We also discuss the potential effects of ketone bodies on intracellular processes such as protein acetylation and post-transcriptional control of gene expression. The exploration of mechanisms underlying the effects of ketone bodies on stem cell function reveals potential therapeutic targets for tissue regeneration and age-related diseases and suggests future research directions in the field of ketone bodies and stem cells.

## Introduction

Stem cells play crucial roles in tissue homeostasis and repair (Li and Clevers, 2010; de Morree and Rando, 2023). These undifferentiated cells possess a unique ability to self-renew and, when needed, replace malfunctioning cells by generating progeny that can differentiate into different cell types. Examples of stem cell models include embryonic stem cells, induced pluripotent stem cells, and adult stem cells (Ramalho-Santos and Willenbring, 2007; Orford and Scadden, 2008).

Adult stem cells are vital for maintaining tissues in the adult body, with their functions adapted to meet the specific needs of different organs. For instance, in gut epithelium and in blood—tissues that undergo rapid cell turnover—stem cells need to continuously self-renew in order to maintain populations of active, cycling adult stem cells. In other tissues, like skeletal muscle, adult stem cells maintain their population size by entering a state of prolonged reversible cell cycle exit called the *quiescent state*. Quiescent stem cells act as a reserve for producing progenitor cells that sustain tissue homeostasis and tissue regeneration in response to injury (Cheung and Rando, 2013; de Morree and Rando, 2023).

**FIGURE 1:**
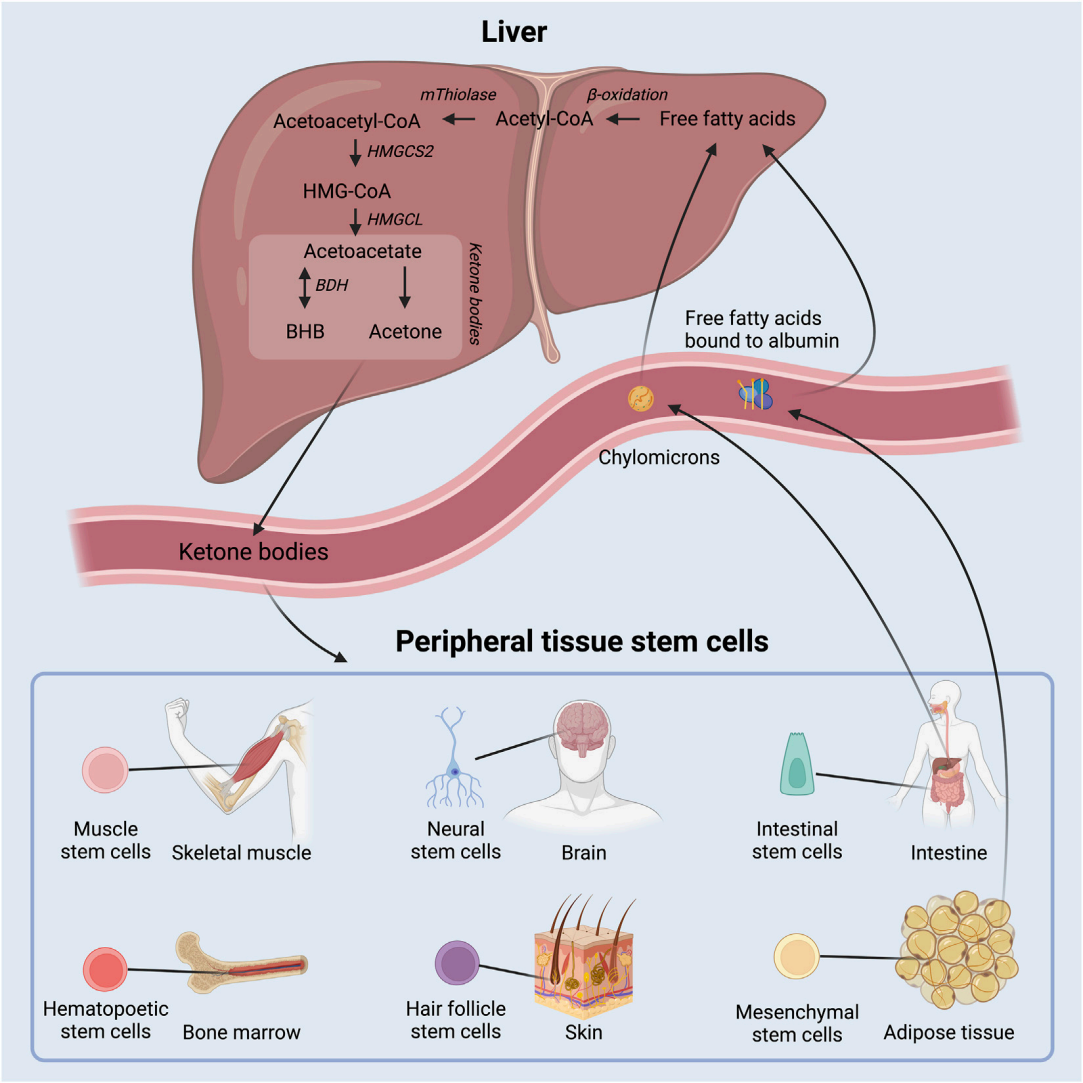
Schematic overview of ketone body production in the liver out of free fatty acids and delivery to peripheral tissue, where they act as energy substrates and signaling molecules. Adult stem cell populations residing in peripheral tissues can take up the circulating ketone bodies. BHB: beta-hydroxybutyrate; HMGCS2: 3-hydroxy-3-methylglutaryl-CoA synthase 2; HMGCL: 3-hydroxy-3-methylglutaryl-CoA lyase; BDH: beta-hydroxybutyrate dehydrogenase.

The regenerative capacity of all organs generally declines as the organism ages and this relates to a decline in stem cell function (Ahmed et al., 2017). Some of the most studied adult stem cells are hematopoietic stem cells (HSCs), skeletal muscle stem cells (MuSCs), neural stem cells (NSCs), hair follicle stem cells (HFSCs), mesenchymal stem cells (MSCs), and intestinal stem cells (ISCs) (Figure 1) (Yi, 2017; Laurenti and Göttgens, 2018; Gehart and Clevers, 2019; Urbán et al., 2019; Relaix et al., 2021). Certain specialized differentiated cell types, such as hepatocytes in the liver, can dedifferentiate and regenerate tissue, giving them a functional resemblance to adult stem cells (Clevers and Watt, 2018). Germ line stem cells in the ovary and testes constitute unique stem cell populations that can give rise to totipotent stem cells after fertilization (de Morree and Rando, 2023).

In recent years, it has become increasingly clear that energy availability can profoundly impact stem cell function, with dietary regimens such as fasting and ketogenic diets exerting notable effects on stem cell biology (Cerletti et al., 2012; Cheng et al., 2014; Tang et al., 2016; Forni et al., 2017; Mihaylova et al., 2018; Long et al., 2019; Mishina et al., 2021; Novak et al., 2021; Terranova et al., 2021; Benjamin et al., 2022). Interestingly, some of the stem cell adaptations to fasting are linked to the presence of ketone bodies (Cheng et al., 2019; Benjamin et al., 2022).

Ketone bodies are water-soluble molecules that contain a ketone-group. They are primarily produced by the liver during low insulin situations, such as fasting, low carbohydrate diets, and prolonged exercise (McPherson and McEneny, 2012). They provide an energy substrate and act as signaling molecules in peripheral tissues (Figure 1) (Puchalska and Crawford, 2017). The presence of ketone bodies in an organism therefore represents certain metabolic situations that may call for a shift in metabolism and cellular functions.

The rapid progression of research into ketone bodies and stem cells may support future treatment regimes in regenerative medicine and age related diseases. Ketogenic diets have been used safely for centuries to treat epilepsy (Barañano and Hartman, 2008; Fei et al., 2020), and currently, both exogenous and endogenous ketone bodies are examined as potential treatment options in numerous diseases including various cancers, diabetes, and Alzheimer’s disease (Feng et al., 2019; Lilamand et al., 2021; Tinguely et al., 2021). In this review we discuss recent studies on cell signaling effects of ketone bodies and discuss aspects that have been observed in adult stem cells and aspects that may apply to adult stem cells. This includes stem cell ketogenesis and uptake, potential metabolic interactions between stem cells and ketone bodies, and cellular signaling pathways affected by ketone bodies.

## Ketogenesis in stem cells

Historically, ketone bodies have been considered energy substrates that could partly substitute for glucose as an energy substrate. This is crucial in cells and tissues that lack the ability to metabolize lipids directly, as is the case with cells of the nervous system, which during ketosis may shift to primarily metabolizing ketone bodies (Owen et al., 1967; García-Rodríguez and Giménez-Cassina, 2021).

Ketone bodies are primarily produced in hepatocytes in the liver at times when circulating insulin levels are low and free fatty acid levels are high due to lipolysis of triglycerides from adipose tissue and chylomicrons. Fatty acids are metabolized through β-oxidation to generate acetyl-CoA, which is then in turn enzymatically converted to the ketone body acetoacetate as depicted in Figure 1. Acetoacetate may be further converted to beta-hydroxybutyrate (BHB) by 3-hydroxybutyrate dehydrogenase and, to a lesser extent, into the byproduct acetone via spontaneous decarboxylation. The concentration of circulating BHB is ∼3 times higher than that of acetoacetate (Balasse and Féry, 1989) and accordingly, most studies of ketone bodies have focused on BHB.

**FIGURE 2:**
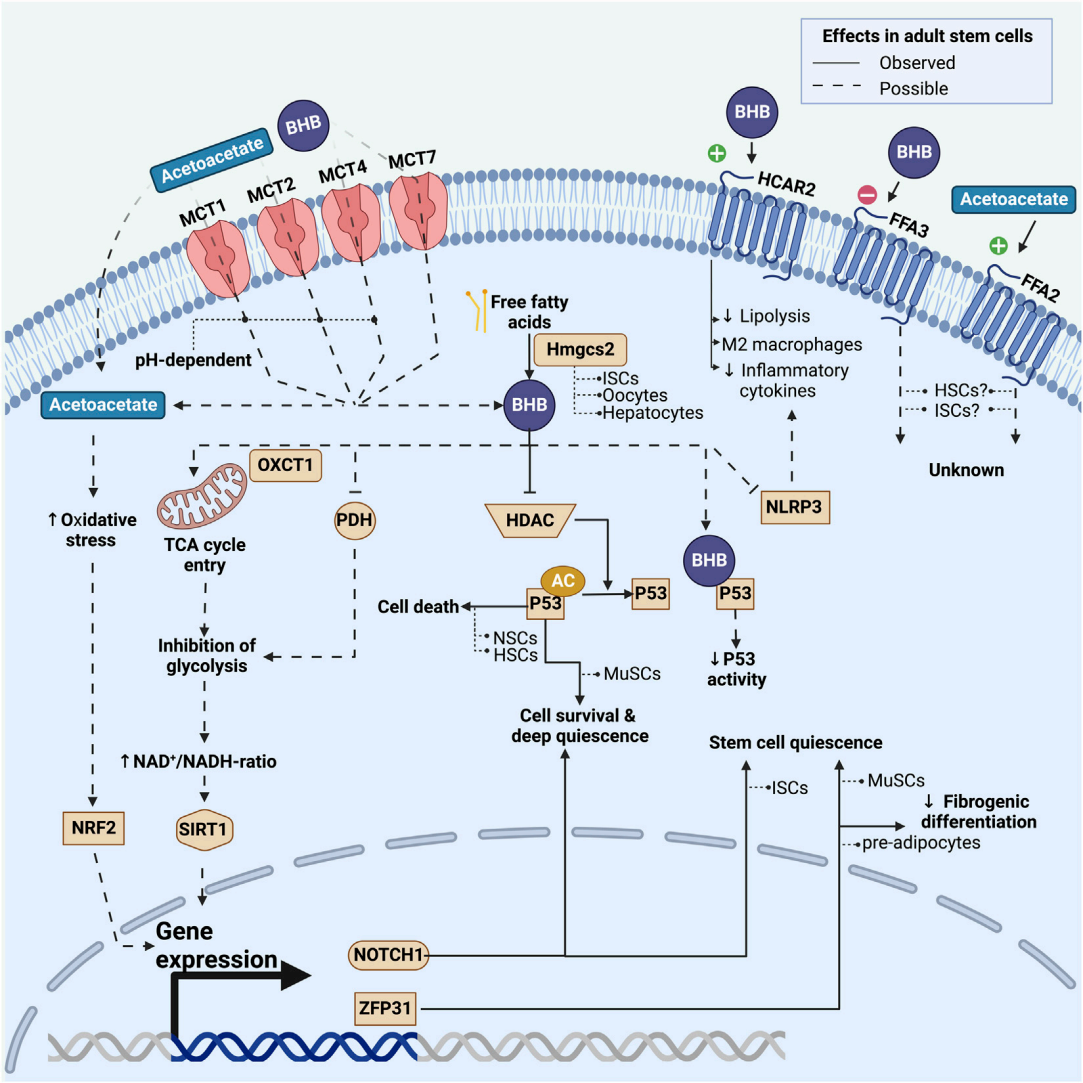
Overview of known and possible mechanisms by which adult stem cells may be affected by ketone bodies. This includes effects on a transcriptional and post-transcriptional level induced by either intracellularly activated pathways or extracellular G-protein coupled pathways. Fully drawn lines represent pathways observed in adult stem cells, whereas dashed lines represent pathways that have been observed in non-stem cell types and could potentially play a role in stem cells. For each pathway we list the stem cell model in which it has been investigated. BHB: beta-hydroxybutyrate; MCT: monocarboxylate transporter; HDAC: class I histone deacetylases; Hmgcs2: 3-hydroxy-3-methylglutaryl-CoA synthase 2; PDH: pyruvate dehydrogenase; AC: acetyl-group; NRF2: Nuclear factor erythroid-2-related factor 2; NLRP3: nucleotide-binding domain and leucine-rich repeat protein-3; MuSCs: skeletal muscle stem cells; NSCs: neural stem cells; HSCs: hematopoietic stem cells; ISCs: intestinal stem cells.

The rate limiting enzyme in the production of acetoacetate, and hence all ketone bodies, is the enzyme 3-hydroxy-3-methylglutaryl-CoA synthase 2 (HMGCS2) (Hegardt, 1999). Few other cell types besides hepatocytes express HMGCS2 (Mascaró et al., 1995; Wang et al., 2016), but interestingly, Lgr5+ ISCs in mice were found to express high levels of HMGCS2 and even to produce ketone bodies (Figure 2) (Cheng et al., 2019). Further, intracellular ketone bodies had functional consequences in the Lgr5+ ISCs as they promoted stemness and regeneration, and limited differentiation into secretory cell types compared to glucose. This finding may suggest that ketone bodies, especially BHB, serve as signaling molecules for the stem cells to conserve energy during fasting or low energy intake situations.

HMGCS2 is also found in germline stem cells in the ovary and testis in rats (Royo et al., 1993) and in the ovaries of neonatal mice (Bagheri-Fam et al., 2020; Niu and Spradling, 2020; Wang et al., 2022). In a germline knockout model for HMGCS2, oocytes in primordial follicles underwent apoptosis rather than adopting the quiescent state - an effect that could be rescued with exogenous BHB (Wang et al., 2022). While HMGCS2 proved important for the number of post-natal follicles, its loss had no measurable effect on fertility (Bagheri-Fam et al., 2020; Wang et al., 2022). HMGCS2 expression also occurs in adipocytes of mice, where, interestingly, expression levels increase when the cells are exposed to ketone bodies. In preadipocytes, however, only negligible amounts of HMGCS2 were found (Nishitani et al., 2022). Future studies will have to confirm the expression of HMGCS2 in human adult stem cells and the presence of ketogenic niches surrounding stem cells in human tissues and to investigate the potential effects of ketone bodies on human stem cell function.

## Ketone body uptake in stem cells

Whereas only a few stem cell types appear able to synthesize ketone bodies, most stem cell types do have the ability to take up circulating ketone bodies from the systemic environment: Acetoacetate is an organic acid that can passively diffuse across the cell membrane (Zou et al., 2016). Whether BHB can do the same remains to be determined. The vast majority of ketone bodies, however, enter the cell by facilitated diffusion through monocarboxylate transporters (MCTs) (Latruffe, 1987). Ketone bodies that are taken up from the systemic environment can enter the tricyclic acid cycle after being converted back to acetyl-CoA (Grabacka et al., 2016).

MCTs are located throughout the body in most cell types where they facilitate transport of a variety of short carboxylic acids (Halestrap, 2013; Iwanaga and Kishimoto, 2015; Pérez-Escuredo et al., 2016). Up to now, MCTs have primarily been investigated in relation to various cancers, where they may play a role in pathogenesis and where expression levels may even be prognostic (Payen et al., 2020). MCT subtypes 1, 2, 4, and 7 (also known as SLC16A1, SLC16A7, SLC16A3, SLC16A6, respectively) can transport ketone bodies, and MCT1, 2, and 4 are proton symporters that are also capable of transporting metabolites such as lactate and pyruvate, with a higher affinity for these metabolites than for BHB (Halestrap, 2013). Few studies have looked at MCT7, and initial results in hepatocytes suggest it also can transport BHB (Hugo et al., 2012; Higuchi et al., 2022).

The role and regulation of MCTs in adult stem cells are not well characterized. Cellular regulation and the micro milieu surrounding adult stem cells will be important to map, especially in stem cells where ketone bodies may affect metabolism or signaling pathways, such as in ISCs and early developing gonads.

## Receptor mediated signaling

Ketone bodies may affect stem cell function by binding to cell surface receptors and affect cell signaling or metabolism (Spigoni et al., 2022). BHB acts as an agonist for the HCAR2 receptor (also known as GPR109A or niacin receptor 1), a G-protein-coupled receptor best characterized in adipocytes, macrophages, and microglia in the central nervous system (Taggart et al., 2005). It can also bind to the free fatty acid receptor 2 and 3 (FFA2 and FFA3) - also known as GPR43 and 41. To the best of our knowledge, these receptors have not yet been studied in the context of adult stem cells. However, cell atlas data reveal robust expression of HCAR2 and FFA2 in LGR5+ stem cells in the gut and in CD34^+^ HSCs in the bone marrow (Tabula Muris Consortium et al., 2018; Tabula Muris Consortium, 2020), suggesting a potential sensitivity of these stem cell types to signaling by circulating ketone bodies.

In adipocytes, BHB-dependent activation of the HCAR2-receptor inhibits lipolysis by decreasing the activity of hormone sensitive lipase (Zhang et al., 2005). Thus, BHB lowers the release of free fatty acids and exerts negative feedback on ketogenesis. In macrophages, activation of the HCAR2 receptor may shift cell differentiation away from the pro-inflammatory M1 phenotype and towards the anti-inflammatory M2 phenotype (Chen et al., 2018; Zhang et al., 2021). In the mouse brain following stroke, activation of the HCAR2 receptor on monocytes reduces inflammation and exerts neuroprotective effects (Rahman et al., 2014). BHB-dependent activation of the HCAR2 receptor on microglia inhibits the production and secretion of proinflammatory cytokines and enzymes (Fu et al., 2015). These anti-inflammatory changes in macrophages and monocytes exposed to ketone bodies may be facilitated through pathways besides the HCAR2 receptor: In mouse macrophages, BHB inhibits the nucleotide-binding domain and leucine-rich repeat protein-3 (NLRP3) inflammasome that promotes the production of the pro-inflammatory cytokines IL-1β and IL-18 (Figure 2) (Youm et al., 2015; Hirata et al., 2021). However, studies in humans have failed to demonstrate changes in cytokine levels during ketosis induced by ingestion of exogenous ketone bodies (Neudorf et al., 2019; Neudorf et al., 2020). Inflammatory responses can change adult stem cell function via proinflammatory cytokines, which has been demonstrated in epidermal stem cells, HSCs, and HFSCs (Xiao et al., 2020; Jahandideh et al., 2020; Caiado et al., 2021; Doles et al., 2012). These results suggest that ketone bodies can affect adult stem cell function indirectly, via altered inflammatory signaling.

The FFA2 and FFA3 proteins are best characterized in the gastrointestinal tract, immune cells, and adipose tissue, where they sense and respond to short-chain fatty acids, such as acetate, propionate, and butyrate (Brown et al., 2003). Early studies failed to reveal a direct interaction between ketone bodies and FFA2 and FFA3 (Le Poul et al., 2003), but later studies revealed that BHB can act as an antagonist of FFA3 (Kimura et al., 2011), and that acetoacetate can activate FFA2 (Miyamoto et al., 2019). This discrepancy could be explained by the use of the physiologically scarce L-isomer of BHB in the earlier kinetic study (Le Poul et al., 2003) instead of the physiologically prevalent D-isomer. The role of FFA2 and FFA3 is not yet fully understood and their expression and function are still to be uncovered in stem cells.

## Metabolic regulation of stem cell function through ketone bodies

Ketone bodies are first and foremost metabolites and could therefore change stem cell function via a change in cellular metabolism. A change in cellular metabolism in stem cells may promote stem cell processes like differentiation or alter stem cell function (Ito and Suda, 2014). For example, disruption of glycolysis in HSCs or glycolytic lactate production in HFSCs resulted in activation out of the quiescent state (Harris et al., 2013; Takubo et al., 2013; Flores et al., 2017).

The rate limiting enzyme in the breakdown of ketone bodies is OXCT1 (Board et al., 2017; Benjamin et al., 2022). While germline loss of OXCT1 is lethal (Cotter et al., 2011), tissue specific loss in adult mice in neurons, myofibers, or cardiomyocytes is not (Cotter et al., 2013). Consistently, the few cases of OXCT1-deficiency (OMIM 245050) did not present any obvious neurological or neuromuscular phenotypes. This suggests that ketone bodies are not an essential energy substrate during homeostasis and that ketone bodies may serve other roles in addition to their role as a metabolite. While OXCT1 is highly expressed in quiescent MuSCs, its deletion did not affect MuSC function nor the effects of exogenous BHB on MuSCs (Benjamin et al., 2022). Whether conditional deletion of OXCT1 affects muscle regeneration or long term muscle homeostasis remains to be determined.

Ketone bodies can substantially modify cellular metabolism, as demonstrated in various tissues. They can decrease glucose uptake in chick skeletal muscle, elevate glycolytic intermediates in human skeletal muscle, and diminish cellular glucose metabolism in isolated rat hearts (Wu and Thompson, 1988; Russell et al., 1997; Cox et al., 2016). Overall, ketone bodies appear to inhibit glycolysis and promote lipid oxidation, which may occur through inhibition of pyruvate dehydrogenase (Ashour and Hansford, 1983). This metabolic shift can increase the cellular NAD+/NADH-ratio (Xin et al., 2018; Elamin et al., 2020) and thereby increase the activity of the NAD+ dependent deacetylase, Sirtuin 1 (SIRT1) as seen in Figure 2 (Newman and Verdin, 2014). SIRT1 is a histone deacetylase and its activation has been linked to maintenance of stemness in HSCs (Matsui et al., 2012) and increased differentiation in NSCs (Hisahara et al., 2008). These findings are in line with findings that link increased glycolysis to higher levels of histone acetylation in stem cells (Yucel et al., 2019).

Dietary interventions are popular among the general public, and ketogenic diets are trending. Such diets inflict major changes on hormonal and metabolic homeostasis (Zhu et al., 2022) and can thereby potentially affect stem cells through pathways unrelated to ketone bodies *per se*. Besides a ketogenic diet, ketosis may arise from other types of interventions, e.g., fasting, prolonged exercise, or exogenous supplementation. Studies that apply mechanistic methodologies to investigate stem cell function directly related to ketone bodies are therefore important.

## Transcriptional and post-transcriptional regulatory effects of ketone bodies

BHB plays an important role in transcriptional regulation, by acting as an inhibitor of class I histone deacetylases (HDACs) (Shimazu et al., 2013; Chriett et al., 2019; Benjamin et al., 2022). HDACs remove acetyl groups from histones, which generally results in a more compact chromatin structure and thereby suppressed gene expression. The inhibition of HDACs by BHB accordingly leads to more open chromatin and increased gene expression. Histone acetylation represents a fundamental regulatory mechanism in determining gene expression, which has been shown to impact the function, differentiation potential, and quiescence of multiple adult stem cell types (Frye et al., 2007; Yucel et al., 2019; Vong et al., 2022; Kueh et al., 2023).

Ketone bodies can also influence the acetylation of soluble proteins. The protein p53 (TP53)—often described as the “guardian of the genome”—plays a pivotal role in maintaining the integrity of DNA and preventing genomic instability (Lambrus et al., 2015; Eischen, 2016). Multiple quiescent stem cell types express p53 at high levels (Meletis et al., 2006; Liu et al., 2009; Liu et al., 2018). In both MuSCs (Benjamin et al., 2022) and hepatocytes (Roberts et al., 2018) p53 can be acetylated and thereby activated by ketone bodies. The protein p53 helps to maintain stem cell quiescence and prevent stem cell exhaustion, thereby serving as a quality control mechanism. Germline deletion of p53, for example, results in increased stem cell proliferation and decreased apoptosis in NSCs (Meletis et al., 2006) and HSCs (Liu et al., 2009) (Figure 2). However, the relationship between p53 and stem cell function is not straightforward: In MuSCs acetylated p53 promotes a deep quiescence and a resilient stem cell state (Benjamin et al., 2022). The importance of p53 for MuSC function is underlined by findings in mice where stabilization of p53 promoted stem cell survival (Liu et al., 2018). The effects of ketone bodies on p53 activation remain an underexplored area in most types of adult stem cells. Considering p53’s varied roles and its specific effects on different tissues and cell types, its interaction with ketone bodies could lead to varied responses in different stem cell populations.

Oxidative stress can impact stem cell function on multiple levels (Lee et al., 2018). Nuclear factor erythroid-2-related factor 2 (NRF2) is a transcriptional factor activated by increased oxidative stress levels to stimulate the transcription of antioxidant proteins (Baird and Yamamoto, 2020; Unoki et al., 2020). In rats on a ketogenic diet, NRF2 is activated (Izuta et al., 2018; Lu et al., 2018), and this regulates stemness, self-renewal, and regenerative capacity in various adult stem cell types, including ISCs, NSCs, MSCs, and HSCs (Dai et al., 2020). Increased NRF2 expression occurs with acetoacetate exposure, but not BHB, in specific cell lines (Figure 2) (Jain et al., 1998; Abdelmegeed et al., 2004; Kanikarla-Marie and Jain, 2015; Shi et al., 2016). The increased NRF2 levels are thought to contribute to long-term antioxidant adaptations during ketosis (Kolb et al., 2021) and could play a role in adult stem cells. The extent to which ketone bodies elevate oxidative stress levels in stem cells remains an open question.

Recently, it was discovered that BHB can be conjugated directly to lysine residues on histones: a process termed beta-hydroxybutyrylation (Xie et al., 2016). This is facilitated by the enzyme p300 (Huang et al., 2021). In addition to histones, beta-hydroxybutyrylation might also occur on the p53 protein, potentially leading to altered p53 activity (Liu et al., 2019). As of yet, findings of beta-hydroxybutyrylation on p53 have been reported for the thymus of fasted mice, in the non-small carcinoma cell line H1299, and in the osteosarcoma cell line U2OS, whereas beta-hydroxybutyrylation on histones has only been demonstrated in the embryonic kidney cell line HEK293 (Xie et al., 2016; Liu et al., 2019). Looking forward, the role of beta-hydroxybutyrylation in stem cells requires further research. Uncovering this could pave the way for the identification of novel therapeutic targets in stem cell biology.

BHB has been shown to enhance the transcription of the ZFP36 gene in cultured mouse adipose mesenchymal progenitor cells, consequently increasing the levels of tristetraprolin protein, thereby inhibiting fibrogenic differentiation (Figure 2) (Lecoutre et al., 2022). Tristetraprolin’s role in inhibiting fibrogenic differentiation could be crucial for maintaining healthy adipose tissue. Notably, tristetraprolin is also important in MuSCs, where it helps maintain the quiescent state (Hausburg et al., 2015). However, whether BHB functions upstream of Tristetraprolin in MuSCs and other adult stem cell models, remains to be determined.

Notch signaling is a highly conserved cell communication system that exists in many multicellular organisms. Notch signaling plays a pivotal role in the fate of different stem cell types where it promotes the quiescent state, thereby maintaining stem cell populations (Bjornson et al., 2012; Mourikis et al., 2012; Wang et al., 2017; Engler et al., 2018). In ISCs in mice, BHB preserved stemness by inhibiting class I histone deacetylases, which in turn increased the expression of NOTCH1 proteins (Figure 2) (Cheng et al., 2019). Given its central role in stem cell biology, Notch may be a target of ketone body signaling across many more stem cell populations.

## Discussion and conclusion

Dietary interventions like fasting and ketogenic diets have gained in popularity among the general public for their potential health benefits. However, these interventions are complex and have widespread impact. In this review, we have highlighted the critical role of ketone bodies, especially BHB, in stem cell biology. BHB is not only an energy source during fasting or low-carbohydrate diets but also a signaling molecule that influences stem cell processes, including quiescence, differentiation, and thereby tissue regeneration.

Interestingly, the outcomes of ketone bodies are variable. For example, in MuSCs, ketone body supplementation induced a state of deep quiescence limiting regeneration, whereas in ISCs, ketone body supplementation delayed differentiation, thereby enhancing tissue regeneration (Cheng et al., 2019; Benjamin et al., 2022). Importantly, this means that ketogenic diets and fasting can have different effects across different tissues. Moreover, this suggests that the metabolic needs for each stem cell model are different and hints at the presence of ketogenic stem cell niches. These findings open up potential new treatment strategies for age-related conditions and tissue regeneration. However, more research is needed to understand the specific mechanisms of ketone bodies on stem cells.

Ketone bodies are far from the only metabolite that is affected by dietary interventions. Potentially, other metabolites can parallel the effects of ketone bodies on stem cells. One candidate is lactate, a product of glycolysis. Like ketone bodies, lactate can affect MuSC function and muscle regeneration (Zhang et al., 2020; Nalbandian et al., 2021). Further, lactate can also be covalently attached to histones in a process dubbed lactylation and thereby affect gene expression (Galle et al., 2022). Whether lactate has dual effects on other stem cell types remains an open question, but these initial findings underscore the importance of dissecting the relationships between metabolites acting as signaling molecules and adult stem cells.
